# Prolonged postoperative urine leakage due to a calyceal diverticulum mimicking a renal cyst: A case report and literature review

**DOI:** 10.3389/fsurg.2022.967525

**Published:** 2022-09-07

**Authors:** Jun-jie Yu, An-qi Yang, Yong-jun Du, Tian-bao Huang

**Affiliations:** ^1^ Department of Urology, Northern Jiangsu People's Hospital, Yangzhou University, Yangzhou, China; ^2^ Department of Urology, College of Clinical Medicine, Yangzhou University, Yangzhou, China

**Keywords:** urine leakage, calyceal diverticulum, renal cyst, conservative treatment, case report

## Abstract

**Background:**

The calyceal diverticulum is a rare cystic cavity that communicates with the collecting system *via* a narrow neck or infundibulum. In clinical practice, part of the calyceal diverticula is difficult to differentiate from simple renal cysts even after contrast-enhanced CT. To date, there have been few kinds of literature works on the diagnosis and treatment of calyceal diverticulum combined with renal pelvis dilatation, especially concerning the treatment of prolonged postoperative urine leakage.

**Case description:**

A 53-year-old woman with calyceal diverticulum and renal pelvis dilatation mimicking a simple renal cyst suffered urine leakage after receiving laparoscopic unroofing of the renal cyst. A persistent urine leakage was observed immediately after surgery, with about 200 ml of drainage fluid per day. We first attempted to place a double-J ureteral stent and indwell a catheter. After failing that, conservative treatment was performed. The core idea of the conservative treatment is retaining the drainage tube for more than 1 month, then clamping the drainage tube for 1 week, and finally removing the drainage tube. By 3 weeks of follow-up, the urine leakage disappeared, and the CT scan showed hydronephrosis of the right kidney without perirenal exudation and the lower pole cyst of the right kidney shrank significantly.

**Conclusion:**

This case, we reported here, is to attract the attention of clinicians. Renal cysts should exclude the possibility of the calyceal diverticulum. If urine leakage is inevitable after surgical treatment, our conservative treatment strategy is also an alternative method.

## Introduction

The calyceal diverticulum is a rare cystic cavity that communicates with the collecting system *via* a narrow neck or infundibulum. The incidence ranges from 0.2% to 4.5% in the general population and around 0.21%–0.45% in patients undergoing intravenous pyelography (IVP) (1–3). In clinical practice, part of the calyceal diverticula is difficult to differentiate from simple renal cysts even after contrast-enhanced CT (2, 4, 5). Urine leakage easily occurs if laparoscopic unroofing of the renal cyst is performed.

To date, most of the literature has focused on treating the calyceal diverticulum. However, there have been few literature works on the diagnosis and treatment of calyceal diverticulum combined with renal pelvis dilatation, especially concerning the treatment of prolonged postoperative urine leakage. Here, we reported a patient with calyceal diverticulum and renal pelvis dilatation mimicking a simple renal cyst who suffered urine leakage after receiving laparoscopic unroofing of the renal cyst and was successfully treated with conservative treatment. We presented the following case in accordance with the CARE reporting checklist.

## Case presentation

**Figure 1:**
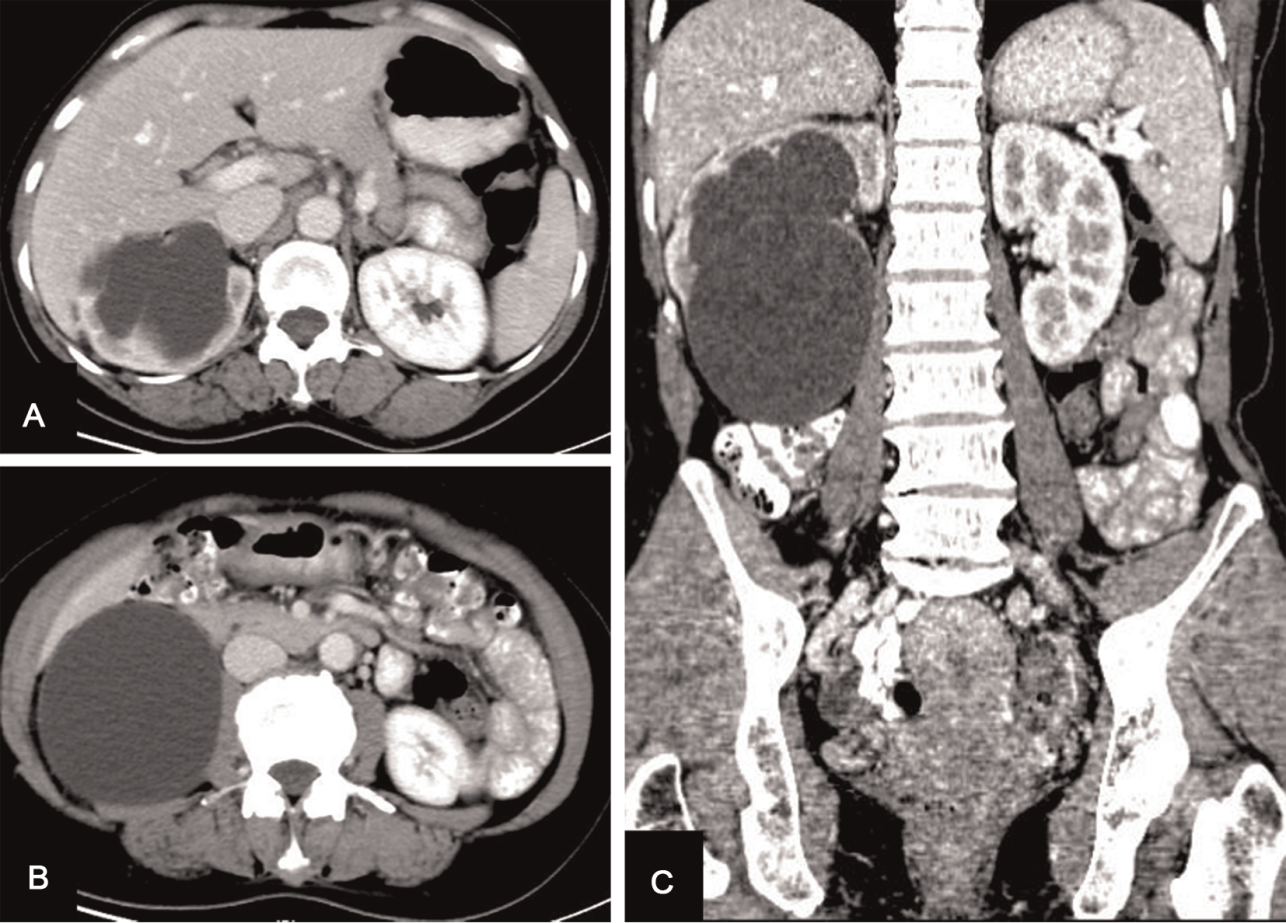
Enhanced CT scan showing a lower pole cyst of the right kidney of approximately 78 mm × 86 mm size and hydronephrosis of the right kidney with reduced renal parenchymal perfusion. (**A,B**) Sagittal images, (**C**) coronal image. CT, computed tomography.

A 53-year-old woman came to our department for a chief complaint of right lumbar discomfort for 3 months. Two months ago, she received sclerotherapy for the renal cyst. No additional discomfort was claimed. B-ultrasonography of the local hospital indicated hydronephrosis and a lower pole cyst of the right kidney. Further contrast-enhanced CT examination was performed, which showed a lower pole cyst of the right kidney of approximately 78 mm × 86 mm in size and hydronephrosis of the right kidney with reduced renal parenchymal perfusion (Figure 1).

After careful preoperative preparation, laparoscopic unroofing of the renal cyst was performed with the agreement of the patient and her family. During the operation, we found a slight deposition of fat around the renal cyst and a thick renal cyst wall. After the excision of the renal cyst wall, we carefully observed and found few indications of a stiffening agent but a small amount of seepage in the base of the cyst, which is not attracted enough attention due to its small volume. However, a persistent urine leakage was observed immediately after surgery, with about 200 ml of drainage fluid per day (creatinine of drainage fluid: 832 umol/l). Then, we attempted to place a double-J ureteral stent and indwell a catheter. Simultaneously, a CT examination was performed, which indicated that the double-J ureteral stent was in place. In addition, from the CT scan, we found that the patient's original hydronephrosis decreased a lot. It is a pity that the patient's urine leakage did not improve in the following weeks.

According to the previous medical history, the intraoperative findings, the prolonged urine leakage, and decreased hydronephrosis in the second CT scan, it was agreed that the patient's preoperative diagnosis should be calyceal diverticulum. Theoretically, once the patient has prolonged urine leakage under this circumstance, open surgery should be carried out earlier because the neck of the calyceal diverticulum is covered with transitional cell epithelium (2), which makes it difficult to heal. However, the surgical treatment also has its disadvantages, such as more difficult surgery resulting from local adhesions in the surgical area and the high risk of collateral injury and bleeding. In addition, the cost of the patient’s treatment will increase accordingly. After weighing the advantages and disadvantages, the patient accepted our preset conservative protocol and provided informed consent. In addition, the conservative procedure was approved by the Ethics Committee of Northern Jiangsu People's Hospital. The core idea of our conservative treatment is to retain the drainage tube for more than 1 month, then clamp the drainage tube for 1 week, and finally remove it. It is noted that the patient's waist symptoms and body temperature should be mainly observed during the clamping period. If lumbar discomfort is obvious or if fever is present, the drainage tube should be opened immediately.

**Figure 2:**
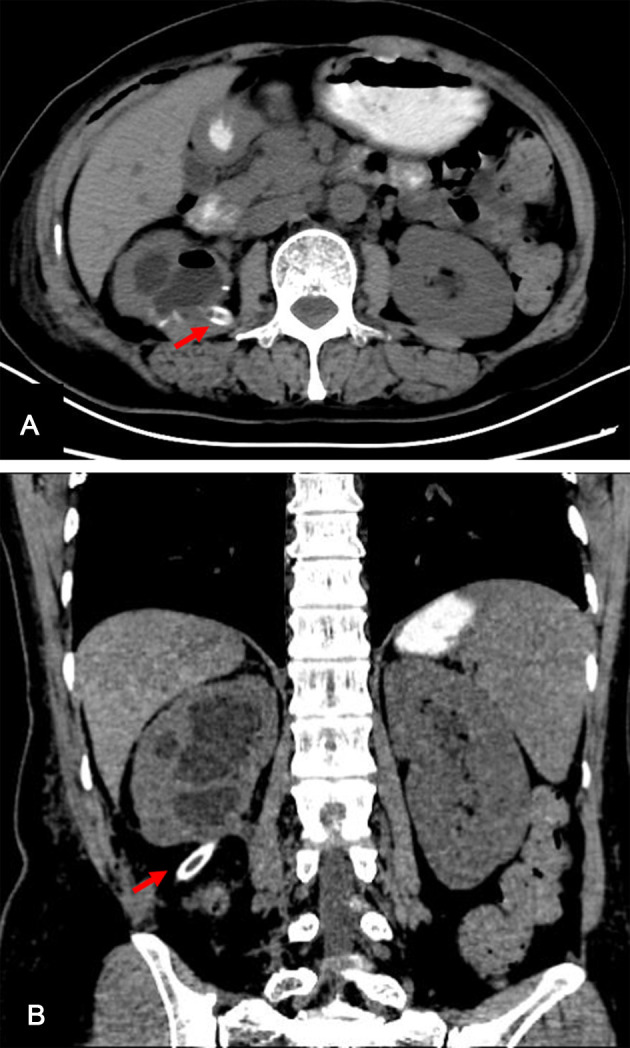
CT scan, which was performed after retaining the drainage tube for 34 days, showing no obvious perirenal exudation. The red arrow refers to drainage tube. (**A**) Sagittal images, (**B**) coronal image. CT, computed tomography.

The patient was hospitalized again about 1 month later. She claimed that the amount of urine leakage remained at about 200 ml per day. Subsequently, the double-J ureteral stent was removed, and the drainage tube was clamped for 1 week. During this period, the lumbar symptoms of the patient were not obvious and the body temperature was normal. Further CT examination showed no obvious perirenal exudation (Figure 2). Then, the drainage tube was removed. Three days later, the patient was discharged.

**Figure 3:**
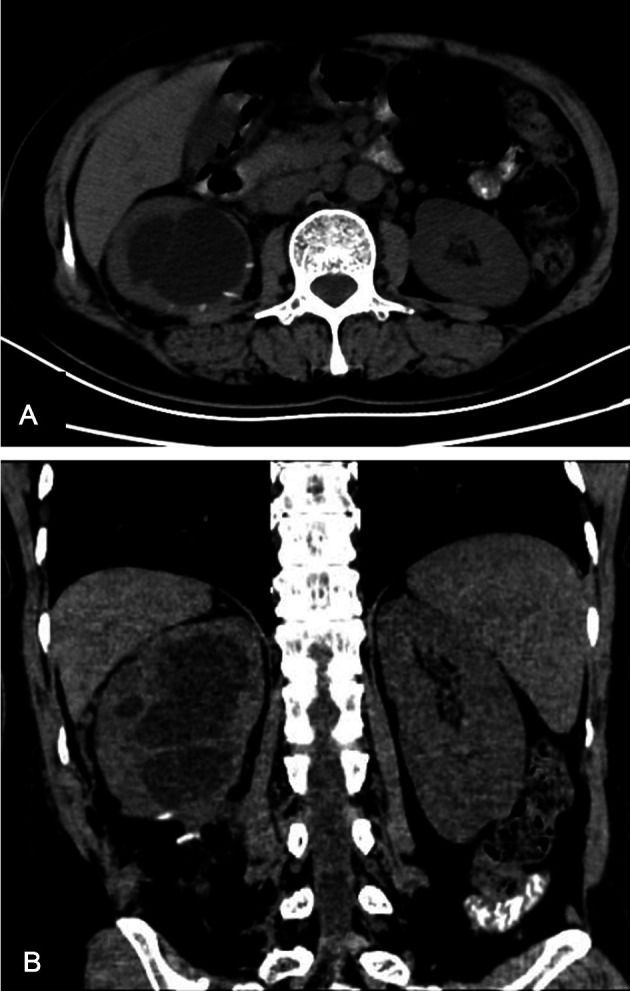
CT scan, which was conducted 3 weeks after drainage tube removal, showing hydronephrosis of the right kidney without perirenal exudation. The lower pole cyst of the right kidney shrank significantly. (**A**) Sagittal images, (**B**) coronal image. CT, computed tomography.

Three weeks after removing the drainage tube, the patient returned to our clinic. Her incision was completely healed. A follow-up CT scan showed hydronephrosis of the right kidney without perirenal exudation, and the lower pole cyst of the right kidney shrank significantly (Figure 3).

## Discussion

The calyceal diverticulum is a rare cystic lesion in the renal parenchyma with a neck or infundibulum between the renal pelvis and diverticulum, through which urine can flow back into the diverticulum. Depending on the location of the diverticulum, it can be divided into two types. Type 1 arises from a minor calyx, while type 2 arises from the renal pelvis or major calyx (6). Calyceal diverticula are often asymptomatic and do not require active surgical intervention (7). Surgical intervention may be considered in these circumstances: (1) the diameter of the calyceal diverticulum is longer than 2.5 cm; (2) the calyceal diverticulum is complicated by stone, infection, or secondary tumor; and (3) the clinical symptoms are obvious. To date, debate still exists over the best surgical treatment for calyceal diverticula, and the choice deeply depends on the location and anatomy of the diverticulum itself (2).

The calyceal diverticulum is easily confused with other pathological conditions on B-ultrasound or nonenhanced CT scans, especially cysts (8). As is known, an enhanced CT scan can improve the diagnostic accuracy of renal lesions, which is routinely used before renal surgery. However, in the present case, enhanced CT did not effectively detect the calyceal diverticulum. As is known, an excretory image is the crucial phase for diagnosing a calyceal diverticula. Three reasons could be taken into account probably. First, the delayed scan time might be too short to achieve the excretory image. Second, the calyceal diverticulum, in this case, was complicated by renal pelvis dilatation, which resulted in poor contrast agent filling in an enhanced CT scan. Third, the neck of the calyceal diverticulum might be narrow, and the contract agent can hardly enter the diverticulum during excretory phase imaging. Hence, prolongation of acquisition is required in highly suspected cases of the calyceal diverticulum, especially in patients with hydronephrosis (9, 10). In addition, a nonenhanced CT scan performed after retrograde urography is a proven alternative way when enhanced CT scan results are negative (4).

According to the previous medical history, the intraoperative findings, the prolonged urine leakage, and decreased hydronephrosis in the second CT scan, it was agreed that the preoperative diagnosis should be calyceal diverticulum. Several points are summarized to minimize the probability of misdiagnosis. First, all renal lesions, especially cysts, should be wary of the possibility of the calyceal diverticulum and needs to be distinguished by enhanced CT with enough scan time. Second, for special cases, such as young giant renal cystic lesions, hydronephrosis with renal cystic lesions, polycystic kidneys, and the like, CT urography should be performed routinely and also a prolonged scan if necessary. Third, for some highly suspected cases without imaging support, a ureteral catheter can be indwelled before surgery and methylene blue injection can be injected intraoperatively to observe the presence of blue staining in the operative area to distinguish the calyceal diverticulum from the renal cyst.

If the calyceal diverticulum is still not diagnosed through the aforementioned methods, and the patient, unfortunately, receives laparoscopic unroofing of the renal cyst, urine leakage will always occur after surgery. PUBMED was searched until April 30, 2022 to identify eligible studies using the following keywords: “renal cyst or calyceal diverticulum or calyceal diverticula” and “urine leakage”. Unfortunately, no relevant study has been published focusing on the management methods and timing of this type of urine leakage. Prolonged urine leakage was mainly reported after partial nephrectomy, which can usually be resolved by indwelling double-J ureteral stent (11). In some rare cases, simple ureteral stenting was insufficient. Several different methods have been described, such as using double ureteral stenting (12), placing a Malecot catheter in the ureter (11), and using glue through percutaneous or endoscopic approaches (13–16). In addition, desmopressin intake combined with ureteral stenting was also proved effective in some cases (17–19). Open surgery for urine leakage is rarely reported in the literature.

Indeed, urine leakage after receiving laparoscopic unroofing of the calyceal diverticulum is different from that after partial nephrectomy, mainly because the neck of the calyceal diverticulum is covered with transitional cell epithelium, which makes it difficult to heal. Theoretically, once a large amount of urine leakage occurs, open surgery should be carried out earlier. According to the intraoperative situation, the neck of the diverticulum should be closed or expanded to relieve urine leakage. The effect of conventional conservative treatment has not been reported before.

In our case, a double-J ureteral stent was indwelled initially but was not effective. Without considering additional surgical intervention, our group adopted a conservative treatment strategy subsequently. The core idea of our conservative treatment is as follows. The first step is retaining the drainage tube until the formation of the sinus tract. The formation time of the sinus tract is based on cystostomy. To our knowledge, the cystostomy's sinus tract can be formed in about 1 month. Hence, we retained the drainage tube for more than 1 month to ensure formation of the sinus tract. After that, the drainage tube was clamped. The sinus tract could protect against large-scale extravasation of urine when clamping the drainage tube. One week later, the drainage tube was removed. By 3 weeks of follow-up, the patient felt comfortable and the incision was healed. It is suggested that conservative treatment is effective for some selected patients.

The benefit of conservative treatment is obvious. It can not only avoid the trauma and risk of the second operation but also avoid the extra costs caused by the second operation, which greatly reduces the occurrence of doctor–patient disputes. Nevertheless, conservative treatment is not suitable for all patients. Conservative treatment is not recommended for patients with fever after clamping the tube or for patients with calculi in the renal pelvis found by CT or B-ultrasound.

Here, we reported this case to attract the attention of clinicians. Renal cysts should exclude the possibility of the calyceal diverticulum. If urine leakage is inevitable after surgical treatment, our conservative treatment strategy is also an alternative method. In our opinion, a conservative treatment strategy should aim at selected patients, comprehensively considering cost, risk, patients’ requirements, and other factors.

## Data Availability

The original contributions presented in the study are included in the article; further inquiries can be directed to the corresponding author.
